# Relationship between immune status after ATG treatment and PNH clone evolution in patients with severe aplastic anemia

**DOI:** 10.1002/jcla.23667

**Published:** 2020-11-28

**Authors:** Honglei Wang, Hui Liu, Ting Wang, Lijuan Li, Chunyan Liu, Liyan Li, Tong Chen, Weiwei Qi, Kai Ding, Rong Fu

**Affiliations:** ^1^ Department of Hematology Tianjin Medical University General Hospital Tianjin China

**Keywords:** cytotoxic T cells, PNH clones, SAA, Th cells

## Abstract

**Objectives:**

To investigate the relationship between immune status and paroxysmal nocturnal hemoglobinuria (PNH) clonal evolution of severe aplastic anemia (SAA) patients who received anti‐human thymocyte globulin (ATG) treatment.

**Methods:**

The clinical data of 102 SAA patients who received ATG were collected and retrospectively analyzed. The remission rate, remission time, response rate, hematopoietic, and immune status were compared. Malignant clones were also observed.

**Results:**

The remission rate of the group with PNH clones appeared after treatment was significantly higher than the group without PNH clones. The response rate at 12 months of the groups with PNH clones was significantly higher than the group without PNH clones. The recovery of Hb and Ret % of patients with PNH clones was earlier than the patients without PNH clones. The reduction of percentage of CD8^+^HLA‐DR^+^/CD8^+^ and Th1/Th2 ratio of patients with PNH clones was both earlier than the patients without PNH clones. Six patients developed myelodysplastic syndromes (MDS).

**Conclusion:**

In SAA patients with PNH clones, the cytotoxic T‐cell function and Th1 cell number recovered more quickly and had better response to IST. A small number of SAA patients with or without PNH clones developed MDS malignant clones.

## Graphical abstract

In SAA patient with PNH clones, the cytotoxic T cell function and Th1 cell number recovered more quickly and had better response to IST. A small number of SAA patient with or without PNH clones developed MDS malignant clones.

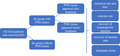

## 1 INTRODUCTION

Severe aplastic anemia (SAA) is an autoimmune disease in which cytotoxic T cells attacking hematopoietic stem cells, characterized by hematopoietic stem cell injury, bone marrow adiposis, and peripheral blood pancytopenia.^1^ A strong association of paroxysmal nocturnal hemoglobinuria (PNH) and AA has been recognized since 1961.^2^ A small population of glycosylphosphatidylinositol (GPI)‐deficient blood cells is often detected in patients with SAA who do not have clinical or laboratory signs of hemolysis. It has been reported that up to 40%–50% of aplastic anemia (AA) patients with initial diagnosis are accompanied by PNH clones,^3^ and PNH clones also often occur in SAA patients after immunosuppressive therapy (IST). Some studies have revealed that the presence of PNH clones is predictive of the response to IST.^4^, ^5^, ^6^ IST prompts improvements of hematopoietic functionalities of the SAA patients by regulating their immune functionalities.^7^ So we retrospectively studied 102 SAA patients treated with anti‐human thymocyte globulin (ATG) plus cyclosporine A (CsA), to investigate the relationship between immune status and PNH clonal evolution after treatment.

## 2 MATERIALS AND METHODS

### 2.1 Patients

We conducted a retrospective analysis in 102 patients who received rabbit ATG plus CsA from January 2006 to January 2016 at the Hematology Department of Tianjin Medical University General Hospital. The diagnostic procedures were compliant with the 2009 British Council for Standardization in hematology aplastic anemia treatment guidelines.^8^ Treatment efficacy was determined based on the Camitta standard published in 1979.^9^

The patients were all detected with complete blood count (CBC), PNH clone, liver and kidney functions, bone marrow smear, bone marrow (BM) biopsy, the immune parameters including Dendritic Cells (DC), T‐cell, Th cell and activated CD8^+^ T‐cell percentage at 0, 3, 6, 12, 24, and 36 months. PNH^+^ was defined as the proportion of CD59^−^ on granulocytes ≥5%. Since the number of counting cells is 50,000, the accuracy of 0.1% cannot be achieved. It takes more than one million to reach the accuracy of 0.1%. The clinical characteristics of all the patients were showed in Table 1. The median follow‐up time was 12 months (1 month–120 months).

**TABLE 1:** Clinical characteristics of three groups before treatment

	The group with PNH clones appeared before treatment (*n* = 22)	The group with PNH clones appeared after treatment (*n* = 10)	The group without PNH clones (*n* = 70)	*p* value
Gender (male/female)	15/7	9/1	40/30	0.1110
Age	23 (6–78)	28 (11–51)	26 (4–68)	0.9559
WBC (×10^9^/L)	1.909 ± 1.572	2.413 ± 1.482	1.735 ± 1.130	0.2903
*N* (%)	27.49 ± 23.54	30.77 ± 18.07	29.22 ± 25.11	0.9451
HGB (g/L)	75.35 ± 13.05	89.11 ± 20.43	74.41 ± 19.10	0.0789
PLT (×10^9^/L)	33.00 ± 17.81	33.80 ± 30.75	27.48 ± 27.25	0.6027
Ret (%)	0.4450 ± 0.4362	0.4350 ± 0.3784	0.4306 ± 0.4811	0.9929
LDH (U/L)	172.5 ± 57.43	176.5 ± 60.50	168.5 ± 70.23	0.7321
CD3^+^CD4^+^/CD3^+^ (%)	19.16 ± 11.59	26.87 ± 7.859	20.06 ± 10.68	0.2533
CD3^+^CD8^+^/CD3^+^ (%)	59.76 ± 19.46	55.85 ± 10.93	53.70 ± 17.38	0.8036
CD3^+^CD4^+^/CD3^+^CD8^+^	0.3719 ± 0.3027	0.4829 ± 0.3162	0.4585 ± 0.3381	0.6971
Th1/Th2	9.550 ± 4.880	10.45 ± 6.975	12.17 ± 10.62	0.8158
mDC/pDC	2.082 ± 2.133	2.456 ± 1.933	2.918 ± 1.140	0.8583
CD8^+^HLA−DR^+^/CD8^+^ (%)	39.90 ± 10.03	36.78 ± 9.03	41.43 ± 10.59	0.8755

No patient had evidence of clinical PNH at SAA diagnosis. So after diagnosis, all patients had received IST including ATG and CsA. The German rabbit ATG with a dose of 3.75 mg/kg body weight was used for a total of 5 days. The dose for CsA is 3–5 mg/kg. All patients were also treated with hematopoietic growth factors (HGFs).

### 2.2 Flow cytometry

Paroxysmal nocturnal hemoglobinuria clones (CD59^−^), T‐cell subset (CD3^+^CD4^+^/CD3^+^CD8^+^), DC subset (LIN1^−^HLA–DR^+^CD11c^+^/LIN1^−^HLA–DR^+^CD123^+^), Th cell subset (CD4^+^IFN‐γ^+^/CD4^+^IL‐4^+^), and activated CD8^+^T cells (HLA‐DR^+^CD8^+^/CD8^+^) were measured by flow cytometry. For Th cell subset, peripheral blood mononuclear cells (PBMCs) were incubated with 50 ng/ml of phorbol ester (Beyotime Biotech, Jiangsu, China), 1 μg/ml of Brefeldin A (Beyotime Biotech), and 1 μg/ml of Ionomycin (Beyotime Biotech) at 37°C for 5 h.

Briefly, fresh PB (400 μl) was collected and separated into five tubes with EDTA‐anticoagulant. A total of 20 μl of mouse IgG1‐PerCP, mouse IgG1‐FITC, mouse IgG1‐PE, mouse IgG1‐APC, and mouse IgG1‐PE‐cy5 antibodies (BD Pharmingen, San Diego, CA, USA) was added into the negative tube. A total of 20 μl of antibody against CD59‐FITC, CD4‐FITC, CD8‐PE, CD3‐PE‐cy5, CD11c‐APC, CD123‐PE, HLA‐DR‐PerCP, LIN1‐FITC, CD8‐PerCP, and CD3‐APC (BD Biosciences, USA) was separately added into different test tubes. Following incubation in the dark at 4°C for 30 min, the red blood cells were lysed with 5 ml of erythrocytolysin solution (BD Biosciences) and subsequently centrifuged at 150 g for 5 min. Following washing, the cells were permeabilized by Cytofix/ Cytoperm Buf Kit (BD Pharmingen) in the dark for 10 min and further washed with PBS. A total of 20 μl of antibody against IFN‐γ‐FITC and IL‐4‐PE (BD Biosciences) was added separately into three test tubes and incubated for 30 min in the dark. Subsequently, the cells were washed twice and resuspended with PBS. At least 300,000 counts were obtained using a BD FACS Calibur cytometer. The results were analyzed by the CellQuest Software (BD Biosciences).

### 2.3 Statistical analysis

GraphPad Prism8 statistical software was used for statistical analysis. Results were expressed as mean ± standard deviations. The independent sample mean comparison had been done using the t test (for data with normal distribution) and nonparametric test (for data without normal distribution). Chi‐square test was used to compare the rates between the groups. A value of *p* < 0.05 was considered statistically significant.

## 3 RESULTS

### 3.1 The clinical data of SAA patients in baseline

A total of 32 patients (31%) had PNH clones, in which the median PNH clones size was 10.57% (5.22%‐42.38%) and the median time was 9 months. Among them, 22 cases with PNH clones appeared before treatment, and the median PNH clones size was 10.35% (6.6%‐28.60%). 18 cases with PNH clones appeared after treatment, among then 4 cases with PNH clones disappeared after treatment. PNH clones appeared in 10 cases after ATG, and the median PNH clones size was 13.55% (5.22%‐42.38%), and the median appearance time of PNH clones was 15 months after ATG.

They were divided into three groups: The group with PNH clones appeared before treatment, the group with PNH clones appeared after treatment, and the group without PNH clones. The three groups before treatment showed no significant statistical significance in white blood cells (WBC, ×10^9^/L), neutrophils (*N*%), hemoglobin (Hb, g/L), platelets (PLT, ×10^9^/L), Ret%, lactate dehydrogenase (LDH, U/L), and immune indexes (*p* > 0.05), as shown in Table 1.

### 3.2 The clinical efficacy comparison of three groups

**FIGURE 1:**
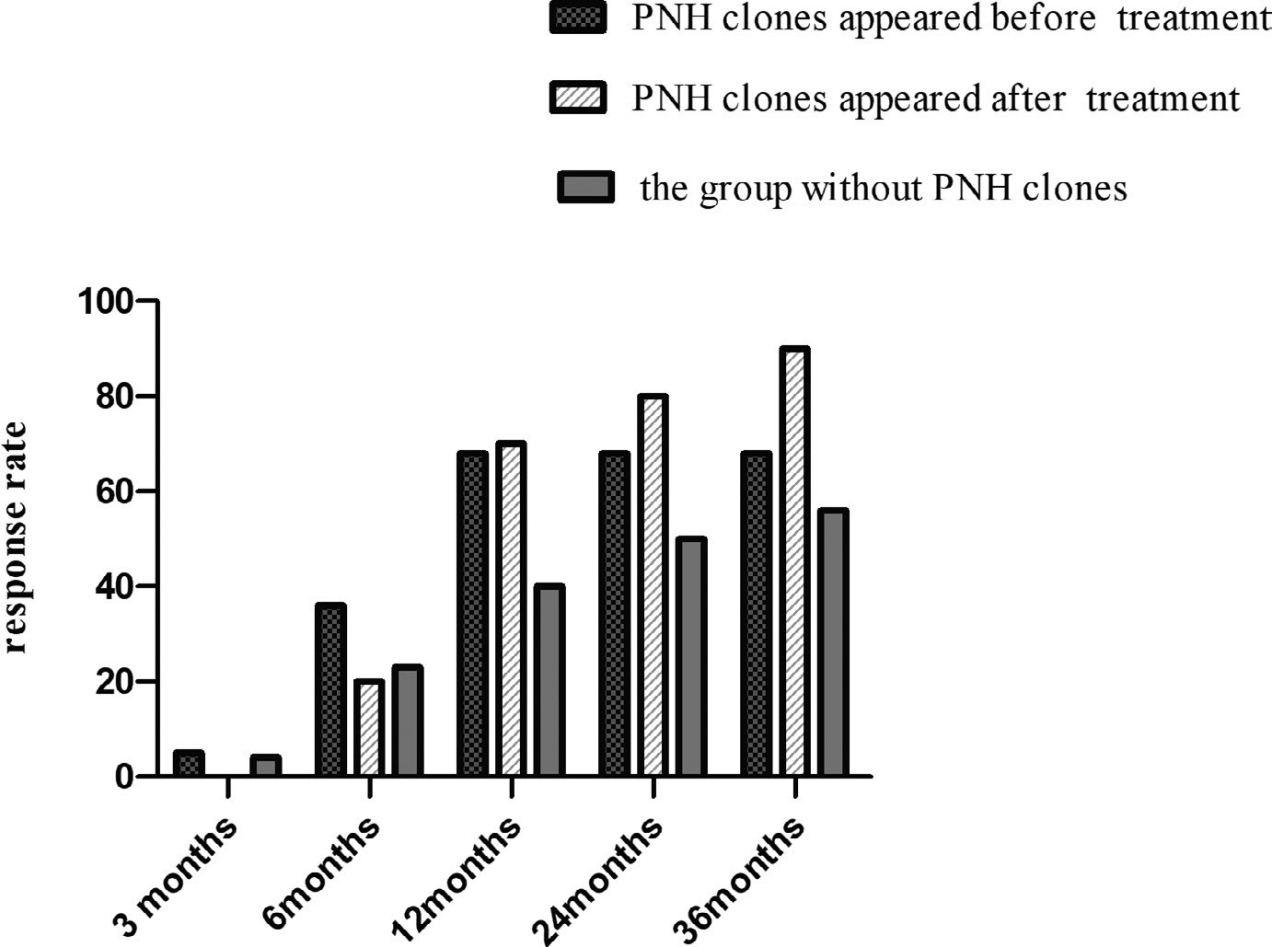
The response rate of three groups at different point times (3, 6, 12, 24, 36 months). The response rate to IST at 12 months and 36 months was statistically significant among three groups (*p* = 0.0349, *p* = 0.0175). The response rate of the groups with PNH clones appeared before and after treatment (68%, 70%) was significantly higher than the group without PNH clones (40%) (*p* = 0.0287, 0.0458) at 12 months. The response rate of the group with PNH clones appeared after treatment (90%) was significantly higher than the group without PNH clones (56%) (*p* = 0.0031). There were no statistical differences in response rate among the three groups at 3, 6, and 24 months after ATG

The remission rates of the three groups were statistically significant (68%, 90%, 56%, *p* = 0.0175), the remission rate of the group with PNH clones appeared after treatment was significantly higher than the group without PNH clones (*p* = 0.0031), but the remission time among these groups was not statistically significant (*p* = 0.1728). Analyzing response rates at different points, the response rate to IST at 12 months was statistically significant among these groups (*p* = 0.0349), the response rate of the groups with PNH clones appeared before and after treatment (68%, 70%) was significantly higher than the group without PNH clones (40%) (*p* = 0.0287, 0.0458). The result of response rate at 36 months was consistent with the remission rate. There was no statistical difference at 3, 6, and 24 months after ATG, which is shown in Table 2 and Figure 1.

**TABLE 2:** The clinical efficacy comparison of three groups

	The group with PNH clones appeared before treatment	The group with PNH clones appeared after treatment	The group without PNH clones	*p* value
Remission rate (CR + PR)%	68	90*	56	0.0175
Remission time (1 month)	9 (3–18)	9 (6–36)	9 (3–36)	0.1728
Response rate (3 months)	5	0	4	0.2847
Response rate (6 months)	36	20	23	0.5626
Response rate (12 months)	68#	70&	40	0.0349
Response rate (24 months)	68	80	50	0.1181
Response rate (36 months)	68	90	56	0.0175

Note

Compared to the group without PNH clones: *p = 0.0031, #*p* = 0.0287, &*p* = 0.0458.

### 3.3 Recovery of hematopoietic function and immune state of three groups (3, 6, 12, 24, 36 months)

At 6 months after ATG, the neutrophil percentage (*N*%) in three groups were statistically significant (*p* = 0.0067), the *N*% of the group with PNH clones appeared after treatment and the group without PNH clones was higher than the group with PNH clones appeared before treatment (*p* = 0.0092, 0.0357). At 12 months after ATG, the Hb in three groups was statistically significant (*p* = 0.0484), and the Hb of the group with PNH clones appeared before treatment was obviously higher than the group without PNH clones (*p* = 0.0155). The Ret% in three groups were statistically significant (*p* = 0.0044), and the Ret% of the group with PNH clones appeared after treatment was obviously higher than the rest two groups (*p* = 0.0289, 0.0019).

At 6 months after ATG, the proportion of CD8^+^HLA‐DR^+^/CD8^+^ in three groups were statistically significant (*p* = 0.0027). The proportion of CD8^+^HLA‐DR^+^/CD8^+^ in group with PNH clones appeared after treatment was significantly lower than that in group without PNH clone (*p* = 0.0057). At 12 months after ATG, the ratio of Th1/Th2 in three groups were statistically significant (*p* = 0.0266), the ratio of Th1/Th2 in group with PNH clones appeared before and after treatment were significantly lower than that in group without PNH clones (*p* = 0.0289, 0.0303). The proportion of CD8^+^HLA‐DR^+^/CD8^+^ in three groups were statistically significant (*p* = 0.0377), the proportion of CD8^+^HLA‐DR^+^/CD8^+^ in group with PNH clones appeared after treatment was obviously lower than that in group without PNH clones (*p* = 0.0024), which is shown in Tables 3, 4, 5, 6, 7.

**TABLE 3:** The hematopoietic function and immune state of three groups (3 months)

Hematopoietic function and immune state	The group with PNH clones appeared before treatment (*n* = 18)	The group with PNH clones appeared after treatment (*n* = 10)	The group without PNH clones (*n* = 55)	*p* value
WBC (×10^9^/L)	7.977 ± 5.863	11.39 ± 7.149	8.390 ± 6.385	0.4139
*N* (%)	65.56 ± 19.85	81.78 ± 9.592	70.14 ± 20.56	0.1589
HGB (g/L)	69.78 ± 21.66	75.38 ± 9.985	75.21 ± 17.91	0.5352
PLT (×10^9^/L)	36.78 ± 47.43	31.13 ± 16.53	31.11 ± 22.95	0.7733
Ret (%)	1.517 ± 1.762	1.905 ± 0.7921	1.672 ± 1.488	0.8284
CD3+CD4+/CD3+ (%)	20.97 ± 11.27	24.98 ± 7.542	21.68 ± 10.24	0.7521
CD3+CD8+/CD3+ (%)	58.27 ± 16.76	53.41 ± 12.25	51.67 ± 15.13	0.5328
CD3+CD4+/CD3+CD8+	0.4534 ± 0.2134	0.4967 ± 0.1523	0.4857 ± 0.2978	0.8214
Th1/Th2	5.408 ± 1.9501	6.443 ± 1.356	6.243 ± 1.798	0.2827
mDC/pDC	2.414 ± 0.8013	2.210 ± 0.6903	2.494 ± 1.367	0.8707
CD8+HLA−DR+/CD8 (%)	31.26 ± 6.312	28.34 ± 5.764	33.45 ± 4.423	0.5216

**TABLE 4:** The hematopoietic function and immune state of three groups (6 months)

Hematopoietic function and immune state	The group with PNH clones appeared before treatment (*n* = 18)	The group with PNH clones appeared after treatment (*n* = 10)	The group without PNH clones (*n* = 55)	*p* value
WBC (×10^9^/L)	7.675 ± 6.596	9.098 ± 4.370	7.149 ± 4.684	0.6409
*N* (%)	58.86 ± 21.51	81.74 ± 8.520	70.29 ± 15.03	0.0067
HGB (g/L)	87.35 ± 30.72	69.75 ± 17.56	78.97 ± 19.75	0.1979
PLT (×10^9^/L)	35.31 ± 28.41	23.88 ± 14.17	41.64 ± 37.58	0.3827
Ret (%)	1.471 ± 1.306	2.216 ± 1.131	1.845 ± 1.464	0.4407
CD3+CD4+/CD3+ (%)	25.97 ± 12.57	21.98 ± 8.339	23.68 ± 11.24	0.7818
CD3+CD8+/CD3+ (%)	51.27 ± 16.76	44.31 ± 13.05	48.67 ± 14.13	0.5452
CD3+CD4+/CD3+CD8+	0.550 ± 0.4634	0.506 ± 0.2212	0.583 ± 0.4970	0.9334
Th1/Th2	3.408 ± 0.9501	3.443 ± 1.183	4.603 ± 2.698	0.4707
mDC/pDC	2.880 ± 1.533	2.175 ± 1.190	1.776 ± 1.901	0.4196
CD8+HLA−DR+/CD8 (%)	22.26 ± 4.993	17.04 ± 6.447^*^	27.41 ± 5.446	0.0027

Note

Compared to the group without PNH clones: *p = 0.0057.

**TABLE 5:** The hematopoietic function and immune state of three groups (12 months)

Hematopoietic function and immune state	The group with PNH clones appeared before treatment (*n* = 18)	The group with PNH clones appeared after treatment (*n* = 10)	The group without PNH clones (*n* = 55)	*p* value
WBC (×10^9^/L)	5.585 ± 1.718	10.72 ± 8.057	6.816 ± 6.373	0.2091
*N* (%)	51.91 ± 17.65	69.29 ± 17.02	55.05 ± 20.12	0.1597
HGB (g/L)	124.5 ± 29.76*	99.43 ± 30.64	93.77 ± 33.71	0.0484
PLT (×10^9^/L)	106.7 ± 75.44	58.57 ± 44.22	73.04 ± 62.25	0.2489
Ret (%)	1.800 ± 0.6279	3.810 ± 3.063**#**	1.378 ± 1.224	0.0044
CD3+CD4+/CD3+ (%)	20.11 ± 9.249	24.24 ± 3.563	24.05 ± 9.830	0.5133
CD3+CD8+/CD3+ (%)	60.56 ± 15.77	40.54 ± 7.592	47.76 ± 15.32	0.0244
CD3+CD4+/CD3+CD8+	0.365 ± 0.1956	0.595 ± 0.2017	0.632 ± 0.4977	0.2413
Th1/Th2	2.070 ± 1.361&	1.758 ± 1.598★	3.210 ± 1.566	0.0266
mDC/pDC	2.737 ± 2.226	2.085 ± 1.731	1.944 ± 1.765	0.6978
CD8+HLA−DR+/CD8 (%)	22.88 ± 17.50	13.72 ± 4.928◆	29.93 ± 7.335	0.0377

Note

Compared to the group without PNH clones: *p = 0.0155, **#** *p* = 0.0019, **&** *p* = 0.0289, ★*p* = 0.0303, ◆*p* = 0.0024.

**TABLE 6:** The hematopoietic function and immune state of three groups (24 months)

Hematopoietic function and immune state	The group with PNH clones appeared before treatment (*n* = 18)	The group with PNH clones appeared after treatment (*n* = 10)	The group without PNH clones (*n* = 55)	*p* value
WBC (×10^9^/L)	4.707 ± 0.7731	5.594 ± 1.714	4.712 ± 1.596	0.5736
*N* (%)	43.00 ± 5.196	49.03 ± 21.86	46.84 ± 15.69	0.8895
HGB (g/L)	135.3 ± 18.04	131.6 ± 7.369	116.8 ± 33.79	0.4696
PLT (×109/L)	160.7 ± 28.29	88.40 ± 25.01	103.2 ± 78.07	0.2835
Ret (%)	2.217 ± 0.7477	1.530 ± 0.7144	1.331 ± 0.7877	0.2734
CD3+CD4+/CD3+ (%)	27.40 ± 16.15	25.43 ± 9.161	30.41 ± 11.24	0.7676
CD3+CD8+/CD3+ (%)	48.38 ± 16.63	37.56 ± 7.556	40.6 ± 9.722	0.2842
CD3+CD4+/CD3+CD8+	0.677 ± 0.5073	0.682 ± 0.2095	0.791 ± 0.3168	0.7967
Th1/Th2	2.188 ± 0.9692	1.644 ± 0.8341	2.368 ± 1.044	0.4260
mDC/pDC	1.704 ± 0.8419	1.965 ± 0.6986	1.616 ± 0.5632	0.7242
CD8+HLA−DR+/CD8 (%)	17.53 ± 12.17	15.70 ± 5.815	18.77 ± 5.676	0.7714

**TABLE 7:** The hematopoietic function and immune state of three groups (36 months)

Hematopoietic function and immune state	The group with PNH clones appeared before treatment (*n* = 18)	The group with PNH clones appeared after treatment (*n* = 10)	The group without PNH clones (*n* = 55)	*p* value
WBC (×10^9^/L)	7.058 ± 4.200	7.340 ± 2.581	7.412 ± 4.079	0.9896
*N* (%)	60.65 ± 13.62	57.23 ± 4.761	52.00 ± 18.83	0.6847
HGB (g/L)	114.3 ± 38.14	141.3 ± 10.07	130.2 ± 37.20	0.5811
PLT (×10^9^/L)	108.0 ± 75.00	118.0 ± 23.43	125.5 ± 81.17	0.9308
Ret (%)	1.903 ± 1.681	2.063 ± 1.372	1.586 ± 0.8151	0.8676
CD3+CD4+/CD3+ (%)	34.58 ± 5.651	23.61 ± 5.707*	34.05 ± 8.111	0.0276
CD3+CD8+/CD3+ (%)	34.38 ± 4.738	39.28 ± 12.47	38.45 ± 7.931	0.5722
CD3+CD4+/CD3+CD8+	1.010 ± 0.1584	0.648 ± 0.2262	0.867 ± 0.2831	0.0707
Th1/Th2	2.056 ± 0.7794	1.904 ± 0.8021	2.218 ± 1.015	0.8236
mDC/pDC	1.360 ± 0.6607	1.140 ± 0.7885	1.185 ± 0.4432	0.8469
CD8+HLA−DR+/CD8 (%)	16.53 ± 9.232	14.09 ± 6.702	17.78 ± 5.376	0.7865

Note

Compared to the group without PNH clones: *p = .0239.

### 3.4 Observation and analysis of malignant clones

All 6 patients had monosomy 7 clonal evolutions and were diagnosed myelodysplastic syndromes (MDS). Abnormal chromosome 7 was found in all patients, including 3 males and 3 females. The median age was 27 (17–43) years. All the chromosome examinations were performed at the initial diagnosis, and all of them were normal chromosome karyotypes. Before evolutions, 4 cases were NR, 1 was PR, and 1 was CR. Among them, 2 patients from the group with PNH clones appeared before treatment, 2 patients from the group with PNH clones appeared after treatment, and 2 patients from the group without PNH clones. The median time from initial IST treatment to −7 abnormality in the 6 patients was 36 (12–75) months. The median follow‐up time was 42 (17–84) months, and the median survival time was 43 (17–84) months. The median time from diagnosis of MDS to death was 9 (5–17) months. 4 patients died during follow‐up, three of the patients died from infection, and one from cerebral hemorrhage. Two patients survived during follow‐up.

## 4 DISCUSSION

Clonal hematopoiesis (CH) was prevalent in aplastic anemia, with CH detected in over two‐thirds of AA patients.^10^ CH is a non‐neoplastic condition that can be associated with diverse genetic alterations, some of which improve cell fitness while others are neutral “passengers”.^11^ Somatic loss of PIGA is the most common manifestation of CH in AA.^12^ AA patients with PNH clones are benign types of bone marrow (BM) failure with immune pathophysiology.^3^, ^13^ The mechanism by which the expansion of PNH cells occurs in AA remains unknown. Various experimental models demonstrated that PNH cells have no intrinsic growth advantage.^14^ One hypothesis is that the PNH cells which can escape autoimmunity have a proliferative advantage over non‐PNH cells by an immune mechanism of selection.^15^, ^16^ The potential mechanisms of immune evasion by PNH cells include the resistance of PNH cells to natural killer (NK) cell and T‐cell activation through the NKG2D receptor due to lack of GPI‐linked cytomegalovirus ul‐16 binding protein (ULBP),^17^ evasion of CD1d‐restricted autoimmune T‐cell attack against the GPI anchor moiety, and reduced rates of apoptosis in GPI‐negative blood cells in the presence of mononuclear cells.^18^ The proliferation of GPI(‐) hematopoietic stem progenitor cells (HSPCs) may not be affected by immune pressure in convalescent patients.^19^ Somatic mutations may also lead to enhanced growth by cooperating with PIGA loss.^20^ Mortazavi Y et al suggested a process of hypermutation in the phosphatidylinositol glycan A gene (PIG‐A) gene in AA stem cells.^21^ In addition, their prognostic role remains controversial. There were studies found that an increase in the proportion of PNH clones cells was correlated with a good response to IST among patients with AA.^22^, ^23^, ^24^ However, there were also reports found that there was no difference between AA patients presented with or without PNH clones, even hematological response of PNH+ group was lower than the PNH group.^25^, ^26^ These discrepancies are probably caused by the lack of a standard threshold for confirming the presence of PNH clones.

A cohort study found that activated CD8+ T‐cell percentage of SAA patients who responded to IST first dipped at 6 months post‐IST, the ratio of Th1/Th2 decreased concomitantly.^7^ Current studies have confirmed that the remission rate of SAA patients treated with ATG combined with cyclosporine can reach 60%–70%.^27^ In our study, patients with PNH clones obtained a high remission rate, especially those with PNH clones appeared after treatment. The remission rate of the group with PNH clones appeared before treatment was 68%, higher than the group without PNH clones (56%), but there was no statistical significance, which was considered to be related to the large deviation of the sample size in this study, and it should be observed after expanding the sample size. At 12 months after ATG, we found that the recovery of Hb and Ret% of patients with PNH clones were earlier than the patients without PNH clones. But the platelet recovery has no significant difference between the three groups, platelet recovered for a long time and very few patients were observed at the later stage maybe the major cause. In terms of immune status recovery, the percentage of activated CD8+ T cells of patients with PNH clones reduced at 6 and 12 months after ATG, Th1/Th2 ratio reduced at 12 months after ATG, both earlier than the patients without PNH clones. This results were consistent with the clinical efficacy analysis of the patients, suggesting that the immune status of SAA patients with PNH clones recovered faster, which was conducive to obtain good clinical efficacy earlier.

It has long been recognized that patients with AA and PNH have an increased risk of developing myelodysplastic syndrome (MDS) and acute myeloid leukemia (AML). Incidence of secondary MDS/AML at ten years can reach 15%–20% in patients with AA ^28^, ^29^ and 2%–6% in patients with PNH.^30^, ^31^ Factors determining an individual patient's risk of malignant transformation remain poorly defined. Factors linked to an increased risk of secondary MDS/AML include disease duration, older age, relapsed/refractory disease, accelerated telomere attrition, and certain genetic alterations.^11^ Of somatic abnormalities in AA, monosomy 7 is most strongly associated with post‐AA malignant transformation. Monosomy 7 has been linked to accelerated telomere attrition.^32^ In this study, the MDS conversion rate of SAA patients was 6%, lower than the reported in the literature, and there were conversion cases in each groups. More patients were needed to see whether there was correlation with the presence of PNH clones.

So in SAA patients with PNH clones, the cytotoxic T‐cell function and Th1 cell number recovered more quickly and had better response to IST. During the follow‐up, a small number of SAA patients with or without PNH clones developed MDS malignant clones. PNH clones should be closely monitored during the diagnosis and treatment of SAA to help evaluate the therapeutic effect and take different treatment regimens. Next‐generation sequencing (NGS) achieves genetic heterogeneity due to the combination of somatic mutations and complex clonal structure, reflecting the sequence of genetic defects, and it is should used widely in these SAA patients for further study of the mechanism of PNH clones and malignant clones.

## CONFLICT OF INTEREST

All authors report no conflicts of interest.

## AUTHORS' CONTRIBUTIONS

Rong Fu designed the research and revised the manuscript. Honglei Wang and Hui Liu performed the experiments, analyzed the data, and wrote the article. Ting Wang, Lijuan Li, Chunyan Liu, Liyan Li, Tong Chen, Weiwei Qi, and Kai Ding contributed to the experimental work and the collection of patients' features. All authors read and approved the final manuscript.

## ETHICAL APPROVAL

Our study confirmed “International ethical guidelines for biomedical research involving human subjects (2002)” developed by Council for International Organizations of MedicalSciences (CIOMS) in collaboration with World Health Organization (WHO) and was approved bythe Ethical Committee of the Tianjin Medical University.

## Data Availability

The data that support the findings of this study are available on request from the corresponding author. The data are not publicly available due to privacy or ethical restrictions.
